# Accuracy of flow measurement with phase contrast MRI in a stenotic phantom: where should flow be measured?

**DOI:** 10.1186/1532-429X-14-S1-P219

**Published:** 2012-02-01

**Authors:** Iman Khodarahmi, Mostafa Shakeri, Melanie Kotys-Traughber, Stefan Fischer, M Keith Sharp, Amir Amini

**Affiliations:** 1 Electrical Engineering, University of Louisville, Louisville, KY, USA; 2 Mechanical Engineering, Univeristy of Louisville, Louisville, KY, USA; 3 MR Research, Philips Healthcare, Cleveland, OH, USA

## Background

Phase-contrast magnetic resonance imaging (PC-MRI) provides a powerful method for the quantification of blood velocity. Accuracy of flow measurement with PC-MRI has been validated with several techniques such as Doppler ultrasound and electromagnetic flowmeters. However, these methods suffer from low accuracy, especially in pulsating flows where short response times are required.

## Methods

Herein, a series of detailed experiments are reported for validation of MR measurements of steady and pulsatile flows with stereoscopic particle image velocimetry (SPIV) on three different stenotic models with 50%, 74%, and 87% area occlusions. Mean inlet Reynolds number was 190 for both steady and pulsatile cases, mimicking the flow of the human common iliac artery.

Axial PC-MRI images were acquired at three sites: inlet (two diameters proximal to the stenosis), throat, and outlet (two diameters distal to the stenosis) using a 3T TX Achieva Philips MRI scanner with slice thickness = 4 mm, resolution = 1 × 1 mm, TE/TR = 3.0/4.0 ms, field of view = 64 × 64 mm, and velocity encoding (Venc) = 30-200 cm/s depending on the imaging section.

**Figure 1:**
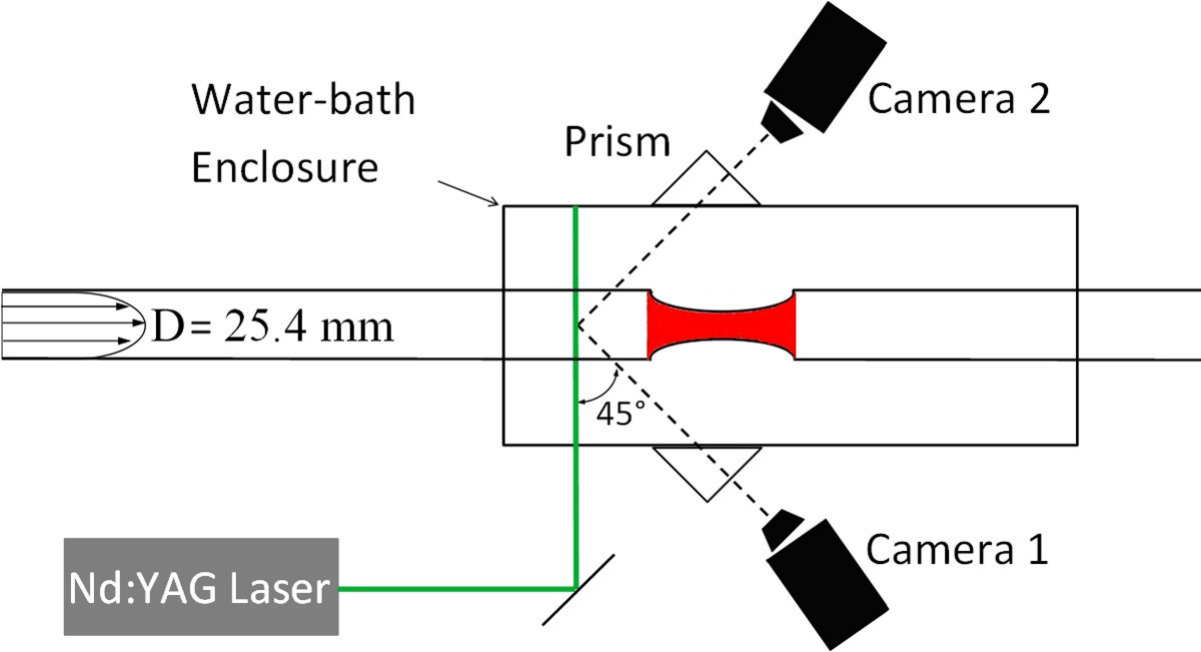
Schematic top view of the SPIV apparatus for flow measurement.

For SPIV purposes, a laser light sheet was passed perpendicular to the axis of the phantom to illuminate the flowing fluorescent particles (Fig 1). A set of image pairs were captured using two cameras looking at the phantom at different angles and the fluid velocity was extracted using a cross-correlation scheme, yielding a nominal spatial resolution of 0.2 mm for the velocity data. The temporal resolution of pulsatile flow measurements was 25 ms, corresponding to 40 measurements per second.

## Results

Agreement between PC-MRI and SPIV was demonstrated for both steady and pulsatile flow measurements at the inlet by evaluating the linear regression between the two methods, which showed a correlation coefficient of >0.99 and >0.96 for steady and pulsatile flows, respectively.

**Figure 2:**
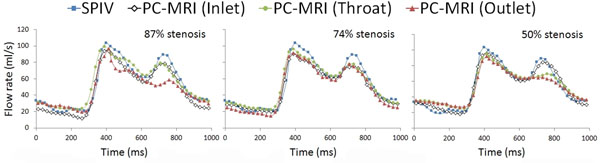
Flow waveforms measured by SPIV and PC-MRI at inlet, throat and outlet for each phantom.

The difference between SPIV and PC-MRI measurements for steady and pulsatile mean flows was less than 5% for both inlet and throat and showed good agreement in all cases (Fig 2). The agreement, however, was weaker at the outlet especially for the 87% stenosis. The flow rate error distal to the stenosis was shown to be a function of narrowing severity.

## Conclusions

Our experiments revealed that the most accurate measures of flow by PC-MRI are found at the throat of the stenosis. This study also illustrates that SPIV provides an excellent approach to in-vitro validation of new or existing PC-MRI flow measurement techniques.

## Funding

This work was supported in part by the National Science Foundation under Grant 0730467 and by an innovative grant from the Clinical and Translational Research Program of the University of Louisville.

